# Evaluating the implementation of a deprescribing intervention in Swiss nursing homes: An observational study using qualitative and quantitative methods

**DOI:** 10.1016/j.rcsop.2026.100713

**Published:** 2026-02-07

**Authors:** Stephanie Mena, Julie Dubois, Florent Macé, Joanna Moullin, Damien Cateau, Marie Schneider, Anne Niquille

**Affiliations:** a Community Pharmacy, Center for Primary Care and Public Health (Unisanté), University of Lausanne, Lausanne, Switzerland; b School of Pharmaceutical Sciences, University of Geneva, Geneva, Switzerland; c Institute of Pharmaceutical Sciences of Western Switzerland, University of Geneva, University of Lausanne, Geneva, Switzerland; d Institute Department of Epidemiology and Health Systems, Center for Primary Care and Public Health (Unisanté), University of Lausanne, Lausanne, Switzerland; e Institute of Family Medicine, Faculty of Science and Medicine, University of Fribourg, Fribourg, Switzerland; f Curtin School of Population Health, Faculty of Health Sciences, Curtin University, Perth, Australia

**Keywords:** Implementation science, Qualitative and quantitative methods, Long-term care, Nursing homes, Deprescribing

## Abstract

Deprescribing, the withdrawal of inappropriate medications, is an appropriate approach to addressing polypharmacy in older adults. However, implementing deprescribing interventions in routine practice remains challenging. This study evaluated the implementation of medication reviews focused on deprescribing, called Individual Deprescribing Intervention (IDeI), in Swiss nursing homes.

Using a hybrid type 2 effectiveness-implementation design, we conducted a qualitative evaluation through semi-structured interviews with nurses, physicians and pharmacists, based on the Framework for the Implementation of Pharmacy Services (FISpH). Quantitative data were collected from administrative sources.

IDeI was successfully implemented in six of seven nursing homes and most healthcare professionals (HCPs) were satisfied. Fidelity was considered good, as only minor intervention adaptations were made. Five main determinants influenced implementation: interprofessional collaboration (facilitator), pharmacist access to medical records (facilitator), motivation fostered by the structured process (facilitator), resident/family resistance to deprescribing (barrier), and resident/staff turnover (barrier). Integration into routine practice was deemed feasible but sustained in only two nursing homes one year later.

IDeI achieved good reach, adoption and HCP satisfaction, providing insights for sustainability. Recommendations include financial incentives, pharmacist training, audit & feedback and greater involvement of residents/families. Findings align with existing literature, emphasizing the need to reinforce interprofessional collaboration and long-term maintenance strategies.

## Highlights

• Intervention was successfully implemented in six of seven Swiss nursing homes.
• Inter-collaboration, access to files, structured processes improved implementation.
• Resident and family resistance and resident and staff turnover were barriers.
• Maintenance one year later achieved only in two nursing homes.
• Financial incentives, pharmacist training, audit and feedback support maintenance.

## 1 Introduction

As in many countries around the world,^1^^,^^2^ the prevalence of polypharmacy (use of five or more regular medications) is high in Switzerland,^3^ particularly in nursing homes,^4^ where it has been estimated to reach 85%.^5^ Polypharmacy in older adults is associated with frailty or hospitalization.^6^^,^^7^ Consequently, the Third Global Patient Safety Challenge, Medication Without Harm from the World Health Organization,^1^^,^^8^ has identified polypharmacy as a key area for action. Inappropriate polypharmacy is especially problematic as it can be defined as medications having a negative risk benefice balance.

Deprescribing, the process of withdrawing inappropriate medications, supervised by a healthcare professional (HCP), ^9^ is considered an appropriate approach to address inappropriate polypharmacy.^10^, ^11^, ^12^, ^13^, ^14^, ^15^, ^16^, ^17^ Anticholinergics, hypnotics and sedatives are examples of drug groups or therapeutic classes targeted by deprescribing initiatives against such polypharmacy in older adults.^18^

However, implementing deprescribing interventions in routine clinical practice remains difficult.^19^, ^20^, ^21^ An implementation science approach is therefore of particular interest as it can help identify the contextual factors that need to be considered to ensure sustainable implementation of these interventions.^22^, ^23^, ^24^, ^25^, ^26^, ^27^, ^28^, ^29^, ^30^ Furthermore, the implementation of deprescribing interventions in nursing homes has not yet been widely evaluated.^23^^,^^31^^,^^32^ In this study, we therefore sought to evaluate the implementation of a deprescribing intervention, named Individual Deprescribing Intervention (IDeI) in nursing homes in two French-speaking Swiss cantons (Vaud and Fribourg). IDeI aimed to deprescribe potentially inappropriate medications (PIMs) through medication reviews. The intervention and results of the effectiveness evaluation, conducted as a randomized controlled trial (RCT), which has proven effective in reducing the doses of PIMs, have been described elsewhere.^33^^,^^34^ To inform future implementation efforts, this article focuses on the implementation evaluation of IDeI which was conducted in parallel with the effectiveness evaluation. More specifically, this study aims to evaluate two implementation strategies (pharmacist training and clinical support) and the following implementation outcomes: adoption, reach, implementation, fidelity, perceived effectiveness and perspectives for maintenance.

## 2 Methods

### 2.1 Ethics approval and consent to participate

The implementation evaluation as part of the IDeI trial, was authorized by the Vaud Cantonal Commission for Ethics in Human Research (decision 2018–01279), as the competent ethics committee. The effectiveness and implementation studies have been prospectively registered on ClinicalTrials.org (NCT03655405) and on the Swiss National Clinical Trials Register (SNCTP000002975), as required by Swiss law. Oral informed consent was obtained from all participants to conduct the interviews and to publish the results as authorized by the Vaud Cantonal Commission for Ethics in Human Research.

### 2.2 Intervention

All participating nursing homes were integrated into an Integrated Pharmacy Service (IPS), which structured collaborations between nurses, pharmacists and physicians since at least 2009. This service is based on quality circles^33^ between the three professions of the nursing homes, where the pharmacist lead the meetings and was therefore already integrated into the NH interprofessional team, without working physically in the nursing home. In addition, all nursing homes involved in IDeI participated in a previous RCT called Quality-Circle Deprescribing Module, which consisted of conducting a specific quality circle session on deprescribing involving pharmacists, physicians and nurses active in a NH, with the aim of producing local deprescribing consensus.^35^

During the preparation phase of IDeI, a 3-day pharmacist training course took place. The operation phase took place between October 2018 and June 2019, in which the pharmacist performed the medication reviews. These were conducted with a structured clinical tool provided by the study team to collect and transcribe data. The pharmacist's proposals concerning the medication(s) to be deprescribed were then discussed within the nursing home team, comprising a pharmacist, one or more nurses and one or more physicians. The resulting deprescribing plan, including monitoring measures, had to be validated by the resident or his/her therapeutic representative. After the first medication change, residents were monitored for four months. This involved documenting and observing the follow-up measures that accompanied the deprescribing and the clinical observation of the residents. All medication reviews were carried out at the same time during the study period. A more detailed description of IDeI is reported elsewhere.^33^^,^^34^ Evaluation of effectiveness, conducted as a randomized controlled trial (RCT), showed that IDeI did not reduce the number of PIMs prescribed to residents, but significantly reduced their dose (incidence rate ratio 0.763, CI95 [0.594; 0.979]), particularly for long-term medications.^34^

### 2.3 Design

To evaluate the implementation of IDeI, we used an observational design employing qualitative and quantitative methods to collect data. The qualitative component consisted of semi-structured interviews with participating HCPs, while the quantitative component involved descriptive analysis of administrative data (see Table 1, Table 2). This study was integrated into an effectiveness-implementation hybrid type 2 study to evaluate implementation and effectiveness in parallel. ^36^

**Table 1:** description, measurement and data source for implementation strategy evaluation.

Implementation strategy	Description	Measurement	Data source
Educational meetings for pharmacists	Pharmacist training	Pharmacists' perception of training	Semi-structured interviews
Ongoing consultation	Clinical support	Number of telephone calls from participating pharmacists for clinical support	Administrative data

**Table 2:** description, measurement and data sources of implementation outcomes.

Implementation stages	Outcomes	Measurement	Data source
Preparation	Adoption	Number of NHs who enrolled to provide the intervention vs. number of eligible NHs	Administrative data
Operation	Reach	Number of NH residents who received at least one deprescribing plan filled by HCPs using the standardized template	Deprescribing plan
	Implementation	• HCPs' experience of deprescribing • HCPs' perceptions of the determinants for the implementation of IDeI • HCPs' satisfaction with the intervention	Semi-structured interviews
	Fidelity	• Description of NH adaptations to the intervention	Semi-structured interviews
	Perceived effectiveness	Perceived impact of intervention by HCPs	Semi-structured interviews
		Relative advantage of IDeI compared with previous intervention led at the NH level (QC-DeMo)	Semi-structured interviews
Sustainability	Perspectives for maintenance	• Proportion of HCPs who intend to repeat the intervention and number of NHs who have repeated the intervention one year later and proportion of HCPs who intend to repeat the intervention • HCP's perception of their ability to integrate the intervention into routine practice • Description of improvements to the intervention proposed by HCPs • Appropriate timing and frequency for conducting a deprescribing-focused medication review • Residents who would benefit the most from a medication review	Semi-structured interviews

IDeI = Individual Deprescribing Intervention, HCP = Healthcare Professional, NH = Nursing home QC-DeMo = Quality Circle Deprescribing Module.

### 2.4 Participants

All participating HCPs, nurses, physicians and pharmacists to IDeI were eligible. The study team recruited them by email at the end of the intervention follow-up. A referring pharmacist was responsible for initiating and coordinating the intervention in each participating nursing home.

### 2.5 Semi-structure interviews

An interview guide, which is presented at Appendix 1, was developed based on the stages of the Framework for the Implementation of Services in Pharmacy (FISpH)^37^ and of the framework Reach, Effectiveness-Adoption, Implementation, Maintenance (RE-AIM).^38^ The FISpH framework was used to design the different stages of the implementation study and ensure that all the components for implementation and implementation evaluation were considered, i.e. (i) factors related to the intervention and levels of context, (ii) implementation strategies and (iii) implementation and (iii) outcomes. The RE-AIM framework was used to structure the implementation outcomes across the stages along with the taxonomy of Proctor^39^ to define additional implementation outcomes. The interview guide was adapted for the three different HCPs (nurses, physicians and pharmacists) involved in implementing IDeI. It was designed to gather participants' perceptions of these different outcomes at each stage of implementation.

Interviews with nurses, physicians and pharmacists were conducted by a PhD pharmacy student (SM) and by an experienced qualitative researcher (JD). More specifically, interviews with nurses were conducted face-to-face in nursing homes and at the pharmacy with pharmacists by SM, as she had more dedicated time to conduct the study. Interviews with physicians were conducted by telephone to facilitate the organization and help the access to physicians. They were realized by JD whose experience overcome the challenge of doing it by telephone. All interviews took place between June 2019 and February 2020. The interviews were conducted in French and audio recorded, after obtaining oral consent from the participants. The transcripts were transcribed verbatim by a research assistant and verified by SM.

### 2.6 Data collection

Two implementation strategies were evaluated according to the categories described by Powell et al^40^ These were pharmacist training and clinical support, that are detailed in Table 1.

Pharmacist training was delivered through educational meetings covering clinical reasoning and realization of medication reviews. Through this training, pharmacists were able to reinforce their clinical, therapeutic and communication skills, aimed at implementing this service in an interprofessional context. This part of the training, which took place face-to-face, was part of a university continuing education program for pharmacists. However, an additional specific course on the conduct of the study was given to participating pharmacists. The study team also provided pharmacists with a structured clinical tool to assist them in performing a medication review and developing a deprescribing plan. The second strategy was to provide clinical support to pharmacists through ongoing consultation. For the duration of the trial, participating pharmacists could call a local pharmaceutical assistance hotline to request pharmaceutical support. This hotline is provided by pharmacists of the university center where the research study took place. The implementation outcomes evaluated in this study are the following: adoption, reach, implementation, fidelity, perceived effectiveness and perspectives for maintenance, which are detailed in Table 2. Most implementation outcomes and strategies were evaluated using semi-structured interviews.

### 2.7 Administrative data

Data to evaluate clinical support were collected through the number of telephone calls received by the clinical support from participants. Adoption was evaluated by collecting data from the number of nursing homes who enrolled to provide the intervention compared to the number of eligible nursing homes.

### 2.8 Data analysis

#### 2.8.1 Semi-structured interviews

Interview transcripts were coded with the assistance of MAXQDA AnalyticsPro software (2022) using thematic analysis^41^ with a primarily deductive approach. Inductive analysis was complementary used in to best capture context-specific elements that were not fully anticipated by the implementation frameworks (FISpH and RE-AIM). First, SM and JD independently coded two interviews, using the interview guide as a basis, then compared and discussed their coding to agree on the codebook. Then, SM applied the codebook to all remaining interviews. Where themes absent from the interview guide were identified, these were coded using an inductive approach. To ensure the validity and rigor of the coding, JD continually cross-checked the codes. Where there was a difference of opinion, the two researchers discussed until a consensus was reached. The codes were then combined into sub-themes and themes. Transcripts from nurses, pharmacists and physicians were first coded together. Themes were grouped according to HCP's type where relevant.

The qualitative data presented in the manuscript are based on quotations from participant. As the majority of our data has been collected through qualitative semi-structured interviews, the COREQ (Consolidated criteria for Reporting Qualitative research) Checklist^42^ was used to write this paper. The satisfaction score of HCPs was obtained orally during semi-structured interviews.

#### 2.8.2 Administrative data

Clinical support was evaluated by calculating the number of telephone calls received by the clinical support from participants. Adoption was evaluated by calculating the number of NHs who enrolled to provide the intervention compared to the number of eligible NHs.

## 3 Results

Six out of seven nursing homes implemented IDeI. In each of them, at least one member of each group of HCPs was interviewed, with the exception of one physician in one nursing home, mentioning time constraints. A total of nineteen HCPs were interviewed in the six participating nursing homes: five physicians, six pharmacists and eight nurses. Interview duration ranged from 18 to 46 min (median: 35 min). In two nursing homes, two nurses were interviewed together, as they were both actively involved in IDeI. The results are presented in accordance with the structure of the outcomes outlined in Table 1, Table 2.

### 3.1 Implementation strategies

#### 3.1.1 Training

The majority of the pharmacists appreciated the training. One pharmacist particularly valued the standardized form provided by the study team for carrying out medication reviews. One pharmacist indicated that he had to use additional resources to carry out medication reviews. Some mentioned that the time required to perform medication reviews was longer than anticipated during training.

#### 3.1.2 Clinical support

None of the pharmacists used the clinical support service during the study.

### 3.2 Implementation outcomes

#### 3.2.1 Adoption and Reach

Eighteen nursing homes were eligible for IDeI and seven agreed to take part (39% adoption rate), four in the canton of Vaud and three in Fribourg. Of these seven nursing homes, one did not implement the intervention as it was unable to recruit residents. A total of sixty-two residents were recruited, thirty of whom were assigned to the control group and thirty-two to the intervention group. Finally, thirty-one residents received a deprescribing plan using the standardized form, as one resident died before initial data collection.

#### 3.2.2 Implementation

##### 3.2.2.1 HCPs' experience of deprescribing

All five physicians interviewed mentioned that they were already performing medication reviews and/or deprescribing prior to the intervention. In addition, two physicians indicated that they performed it in collaboration with nurses or pharmacists.

Phys_NH1: 'So yes, we used to do that (…) Once a month, really, I go through everything (…), each time I check whether the medications that are there still seem useful or not, and depending on that, I also do biological checks to see if such and such a thing is still indicated or not'.

Additionally, one nurse indicated that she had previously identified residents for a medication review and recommended it to the physician during the medical visit without specifying the criteria she applied.

##### 3.2.2.2 HCPs' perceptions of the determinants for the implementation of IDeI

This section presents the determinants for implementing of IDeI, classified by themes related to the healthcare professions and residents/families. A summary of the determinants and illustrative quotes are presented in Appendix 2.

• HCPs related factors

For some HCPs, mainly nurses and physicians, certain drug classes were considered more difficult to deprescribe, either due to the residents' reluctance (e.g. sleeping pills) or because they were perceived as essential by HCPs (e.g. cardiovascular medications). Nurses mentioned the use of alternative therapies to replace medications. One mentioned the physician's belief in these therapies as a facilitator. Other HCPs noted that the study's structured process had motivated them to undertake deprescribing.

Interprofessional collaboration was considered by several HCPs as an essential foundation and facilitator for deprescribing in general. Several nurses and one physician felt that that the involvement and assistance of pharmacists in the process proposed by IDeI was useful for deprescribing. Some physicians and one pharmacist saw an advantage in the involvement of nurses, particularly in facilitating information and collaboration. While collaboration was judged as good by most HCPs, some felt that it needed to be improved. Indeed, one pharmacist pointed out that he had missed the opportunity to collaborate with the nurses and would have liked to have more contact with them in the preparation of the medication review. As physicians are responsible for residents' treatment, some nurses and pharmacists stressed the need to obtain their approval in the deprescribing process, which was seen as a key factor in the successful implementation of the intervention. In addition, the fact that the three groups of HCPs worked in the same place was also considered useful by one physician, as it facilitated communication between them.

• Nurses related factors

According to the nurses, one of the main barriers to implementing IDeI was the high turnover of nurses. One pharmacist indicated that nurses feared deprescribing, because of the potential negative effects on residents. One of the facilitating factors mentioned by the pharmacists was the nurses' knowledge of the residents. The nurses also stressed that this was a determining factor for all HCPs in implementing deprescribing.

• Pharmacists related factors

Lack of easy access to residents' medical records, as well as lack of general knowledge about residents, were also seen as obstacles for pharmacists. Some pharmacists indicated that experience was needed to perform medication reviews.

• Physicians related factors

Physicians reported some resistance to deprescribing among some of their colleagues because it represented a challenge to their practice. One physician considered external physicians, i.e. those who care for only a few residents and visit them only occasionally, as a barrier as opposed to attending physicians, who are responsible for the majority of residents and visit them more regularly. Physicians mentioned their training in psychogeriatrics and the coordination between physicians in a nursing home as facilitators. The latter was recognized as enabling better follow-up of residents.

• Residents/families related factors

For some HCPs, mainly nurses and pharmacists, family involvement was seen as a difficulty in the decision to deprescribe, because they were not interested, difficult to contact or not involved. However, nurses also cited residents' and families' trust in the nursing team as a factor facilitating deprescribing.

Some HCPs emphasized the refusal of residents/family to be deprescribed medication.

The high rate of resident turnover, the low number of residents complaining about over-medication and the placebo effect of residents, i.e. the fact that the physicians and nurses perceived that some residents were convinced that they were actually benefiting from an effect among some medications objectively eligible for a deprescribing, were perceived as barriers by HCPs. Indeed, they reported that a a high resident turnover rate made it more difficult to implement deprescribing.

One physician found it helpful for the family to know the resident's previous experience of deprescribing, as it would assist them in understanding the various approaches that had been attempted in the context of deprescribing. Another physician found it easier to deprescribe when he reassured the resident about deprescribing, or when the resident had lost the capacity for discernment. He drew an analogy with the pediatric population, explaining that in the case of residents lacking the capacity for discernment, it's up to the physician to determine what's most appropriate for them. Conversely, one physician argued that residents capable of discernment were more aware of their treatment and therefore more inclined to accept the deprescribing of a medication. This view was also shared by a pharmacist and two nurses, who felt that it is or would be easier to deprescribe when residents are still capable of discernment.

#### 3.2.3 Satisfaction with the intervention

Most HCPs were satisfied with IDeI, with a mean satisfaction score (± standard deviation) out of 10 of 8.4 (±0.8) for nurses (*n* = 7/8), 8.5 (±0.6) for physicians (*n* = 4/6) and 7.7 (±1.0) for pharmacists (*n* = 6/6). Scores ranged from 6 to 10.

#### 3.2.4 Fidelity

##### 3.2.4.1 Adaptations

Three of the six nursing homes mentioned four adaptations to IDeI, which are as follows:

(1) No communication of treatment changes to the resident:

Pharm_NH7: 'We had the idea of discussing proposed changes with the resident, but this was never done. So it was the physician who decided to stop (…). He [the physician] immediately accepted the proposals, and immediately changed the treatment without taking the next step, which would have been to ask the residents or their families. This was the case for all residents for whom treatments were modified'.

(2) Active deprescription by the physician for all residents, i.e. not only the intervention group but also the control group and other residents, and thus potentially the control group:

Nurse_NH3: 'because, in fact, we deprescribed (…) many other [residents], you know, during the same period; it's just that we didn't have the consent forms'.

(3) Training more nurses to perform IDeI (e.g., one nurse for each floor of the nursing home):

Nurse_NH6: 'In this institution, we decided (....) that the choice of residents (…) was made by the UHN,i.e. the unit head nurses, because we didn't have enough time to explain it to all the graduate nurses. We, the UHN, also work as nurses in the care sector, so participation was not a problem. And that meant five units, five people, to whom I had to explain the study, instead of fifteen people. So we made this choice to speed things up a bit'.

(4) Residents' life goals not considered when defining the resident-centered deprescribing plan. In four out of six nursing homes, HCPs were unable to take the residents' life goals into account when defining the deprescribing plan, either because they did not have access to them (4′), or because the residents' capacity for discernment was insufficient (4″).

(4′) Pharm_NH7: 'I didn't have access to the [life] goals that were clearly defined and then I couldn't adapt the [treatment] recommendations based on that [life] goals. (…) We had this idea that we had to discuss the proposals for change with the resident, this was absolutely not done'.

(4″) Nurse_NH4: 'So we decided not to look specifically at [life] goals'.

#### 3.2.5 Effectiveness

##### 3.2.5.1 Perceived impact of intervention

Varied experiences were observed with regard to the perceived impact of the intervention. Indeed, several HCPs, particularly nurses, reported that the intervention had little impact, resulting in deprescribing of fewer drugs than expected. However, some of them felt that the intervention was still beneficial for the residents. More specifically, the main positive aspects concerned the beneficial impact on residents' health (1) and the fact that deprescribing can have a positive impact on the healthcare system by reducing healthcare costs (2). One nurse reported that she had observed neither deterioration nor improvement, but that the resident was calmer (3).

(1) Nurse_NH2: 'It was good for them because we realized that when people did well afterwards, there were no cases where we had to go back on deprescribing; in most cases, we could really go all the way. That's why I think the patient aspect was also beneficial for them'.
(2) Phys_NH3: 'it was something easy, and that it has an interest for my patient in the first place, and also for health economics in the second place. It was both useful in a way and motivating'.
(3) Nurse_NH3: 'We did reduce one of the medications, (…) we didn't see any deterioration, which is already good, but we didn't see any improvement, except that, in recent months, he finally agreed to stay with us, he's much calmer, he insists less on going home'.

On the other hand, a nurse mentioned that the intervention had resulted in more medications being prescribed than before the intervention, due to their physician's resistance to deprescribing (4). One pharmacist estimated that the intervention resulted in fewer drugs being deprescribed than expected (5). Other HCPs reported that some reintroductions had taken place during follow-up (6).

(4) Nurse_NH2: 'we were frustrated, (…), we realized at the end that we had done this study for nothing, given that now, if we analyze the results, (…), instead of deprescribing, I think there are even more drugs than at the beginning of the study'.
(5) Pharm_NH6: 'in the end we realized that we could only touch on a few things (…) we started with the idea that we were going to make an extraordinary deprescribing on treatments of twelve thirteen drugs, and then in the end we affected one, two or even three at the most'.
(6) Pharm__NH6: 'And then, over time, you realize that you're going to have to reintroduce something very quickly; of course, we first tackled proton pump inhibitors, for example, but very often we had to reintroduce them'.

##### 3.2.5.2 *Relative advantage of IDeI* vs *QC-DeMo*

When comparing the two consecutive deprescribing interventions (QC-DeMo and IDeI) carried out in nursing homes, some HCPs appreciated the medication review approach (IDeI) more than the QC-DeMo approach and vice versa, for different reasons. Most HCPs felt that the medication review approach (IDeI) was more useful for deprescribing than the quality circle approach (QC-DeMo). One pharmacist said that the IDeI was more appropriate because the intervention took place at resident level (1), a point supported by a nurse who pointed out that improvement at resident level was easier to observe (2) and by a physician who found it much more useful because it was much more targeted (3).

(1) Pharm_NH6: 'So it's clear to me that IDeI seemed more useful, more relevant and more coherent, insofar as we were really going to make an analysis (…) adapted to the resident'.
(2) Nurse_NH2_2: 'I think it's very important to do the second [IDeI] intervention just to see if there's an improvement [in the resident] or not (…) but I think there's a definite improvement [in the resident], either way. So I think it's important to do the second [IDeI] intervention as well'.
(3) Phys_NH1: 'So I would say that the second [IDeI intervention] is much more useful, because it's much more targeted. I think if you want to make (…) more cost-effective for everybody, (…) I would say that in terms of health (…) it's the second [IDeI intervention] that's better because it allows you to target each case individually'.

One physician and one pharmacist felt that it was more appropriate to repeat the QC-DeMo intervention rather than apply the IDeI intervention. One of the reasons given by the physician was that QC-DeMo was well suited for the practice in a nursing home setting. As for the pharmacist, he also found that he was making good progress with QC-DeMo, as he was able to extract treatment data by resident, if necessary, from the drug class treated during the quality circle.

#### 3.2.6 Maintenance

##### 3.2.6.1 Proportion of HCPs who intend to repeat the intervention and number of NHs who repeated the intervention one year later

During interviews, most HCPs expressed their intention to repeat the IDeI intervention. Specifically, three out of five physicians wanted to repeat it. One physician specified that it should be included in the medical visit so that it could be implemented in routine practice. The reasons why the two physicians did not intend to continue were: retirement for one, while the other indicated that he intended to continue deprescribing, but through the quality circle of the Integrated Pharmacy Service (IPS). All but one pharmacist intended to repeat IDeI, due to lack of remuneration. Every nurse interviewed also expressed the intention to continue the intervention. One nurse specified that the agreement of physicians was essential to maintaining it and that increasing awareness about deprescribing among physicians was paramount. However, we observed, through monitoring of the IPS that IDeI was maintained in only two nursing homes one year after the intervention.

##### 3.2.6.2 Improvements

Most HCPs suggested improvements following the study. The most frequently cited were: a better repartition of tasks between the three groups of HCPs to carry out the intervention (1), extending the intervention to all nursing home residents (2), spreading medication reviews over the year rather than carrying them out all at once (3), the implementation of long-term monitoring of prescribed medications by the medical and nursing team and transmission to the pharmacist (4), the planning of an annual interprofessional meeting to review the treatments of all residents, or more frequent interprofessional meetings (5).

(1) Phys_NH2: ′Most of the time, these are established physicians who have other activities on the side. I think it's good that the work is shared. (…) I've heard that the nursing profession also has a huge amount of administrative work, and that the physician has no less. So (…), the pharmacist, I don't know how, but let's just say that for this kind of study, it has to be shared to some extent'.
(2) Phys_NH3: ′For me, the improvement lies in broadening the scope of action; in other words, all patients living in the nursing home can be included in this approach because it's more a medical ethical vigilance approach, it's in no way an abuse approach'.
(3) Phys_NH3: ′Clearly, if we were to adapt this study to nursing home practices, well, we'd spread this kind of deprescribing out over the whole year, at the rate of one or two residents a week'.
(4) Pharm_NH2: 'I think that if you want to deprescribe, you have to do a very strict follow-up, because otherwise you'll never get there. Because I mean, I think once a month, we should follow these patients to see where we're at'.
(5) Nurse_NH7: ′Maybe we should see each other more; personally, I mean, between physicians, pharmacists (…), I think that would be a proposal. We met twice, I think, no more, during the study'.

Two other elements linked to nursing home practice were also highlighted by HCPs: the organization of ongoing training for HCPs in geriatrics, drug knowledge, literature updates (6), and the participation of a pharmacist (clinical or community) in the medical visit in all nursing homes (7).

(6) Nurse_NH1: 'Basic training for caregivers. (…). Maybe it would be interesting to have some modules in the training program, in pharmacology (…) knowledge of substances and side effects of drugs, but also, interactions between them'.
(7) Phys_NH2: ′ (…) it is highly desirable for every nursing home (…) to work with a pharmacist or clinical pharmacist and therefore visit once a week, twice depending on the size of the facility, but it seems necessary to me'.

##### 3.2.6.3 HCP's perception of their ability to integrate the intervention into routine practice

Most HCPs felt that the intervention was feasible, acceptable and could be integrated into their usual practice.

Nurse_NH6: 'So it won't be an extra workload, but given the approach we've taken, I think it will be quite possible to integrate it into our future; it won't take us any longer'.

One pharmacist noted that the study workload was low, as the nursing home team was already performing medication reviews prior IDeI. However, most pharmacists indicated that their workload was significant. One pharmacist pointed out that this work should be remunerated as part of routine practice.

Pharm_NH2: 'It's a job that needs to be paid for, because really, otherwise the pharmacist can't do it as part of his job'.

Several nurses also found it difficult to find the time to devote to the procedure. Four of them even mentioned that they had to do it in their spare time. They therefore asked whether it would be possible to integrate it into their working hours in the future.

Nurse_NH2: 'I couldn't do it in my working time, so I had to do it in my free time (…), it would be good, as far as possible, to integrate it into working time'.

##### 3.2.6.4 Appropriate timing and frequency for conducting a deprescribing-focused medication review

HCPs opinions differed regarding the ideal time to carry out a medication review. They suggest that it be carried out during the medical visit, when the resident enters the nursing home, or on discharge from hospital. Other HCPs suggest waiting one to six months of clinical and behavioral observation to get to know the resident better, before performing a medication review.

In terms of appropriate frequency, most HCPs mentioned that medication review should be carried out once a year, while some nurses and pharmacists also mentioned that it should instead be carried out once a month, every four months or every six months. One pharmacist indicated that the frequency depended on each resident's state of health, but that it should be at least annual for all.

##### 3.2.6.5 Residents who would benefit most from a medication review

According to several HCPs, mainly physicians and nurses, the residents who would benefit most from a medication review are: multimorbid residents or those whose condition is unstable, those who have the highest number of medications and/or those who want to use as few medications as possible. One physician also felt that all nursing home residents could benefit from a medication review.

## 4 Discussion

Our results showed that the intervention was well implemented in terms of reach, adoption and HCPs satisfaction, with significant input from participants for future maintenance of the intervention.

The main facilitators of deprescribing identified by HCPs are interprofessional collaboration and structured processes that motivate HCPs to carry out the intervention, which is consistent with the existing literature.^43^^,^^44^ Interprofessional collaboration has already been shown to be a key element in the successful implementation of complex interventions.^20^^,^^45^, ^46^, ^47^ Although it is part of the IDeI, we observed that it could be further strengthened. Other interesting facilitators were reported by HCPs such as residents' trust in nurses, the family's knowledge of the resident's, previous experience of deprescribing, nurses' knowledge of residents^48^ and the use of alternative therapies to replace medication. Interestingly, residents' capacity for discernment was seen as both a barrier and a facilitator by HCPs. A study has been published by the Swiss Academy of Medical Sciences on this subject, to evaluate the challenges and current practice regarding residents' capacity for discernment.^49^ They also highlighted the importance of raising awareness among HCPs through better education and training. A guideline has also been developed. It defines the principles to be respected and describes the assessment procedure in general and in some specific medical fields (e.g. palliative care).^50^

The main barriers identified to the implementation of deprescribing are consistent with the literature, including the difficulty of involving family,^13^^,^^51^, ^52^, ^53^ resident/families resistance to deprescribing,^20^^,^^26^^,^^54^ fear of the consequences of deprescribing by HCPs and residents,^22^^,^^55^^,^^56^ high nurse turnover,^57^, ^58^, ^59^ and high residents turnover.^60^ These barriers should be directly targeted by implementation strategies for future implementation efforts by e.g. involving more conversations with the residents/families.^48^^,^^61^^,^^62^ In addition, lack of experience and continuing education are barriers cited by HCPs to implementing deprescribing interventions, as highlighted by previous studies.^45^^,^^63^ Pharmacists cited their lack of familiarity with residents as a barrier to their selection for deprescribing, another element identified by other researchers.^46^^,^^64^^,^^65^ This highlights the need to strengthen interprofessional collaboration between nurses, whose level of knowledge of the resident is high, and pharmacists. The difficulty caused by external physicians in nursing homes, i.e. physicians who look after only a few residents, compared with attending physicians, has already been highlighted in the same context by Foley et al^56^

According to HCPs, the residents who could benefit most from a medication review are in line with current clinical guidelines, which recommend targeting in priority multimorbid residents or those with the highest number of medications.^66^^,^^67^ However, the notion of unstable patients has not been clearly defined by the HCPs. This should therefore be further evaluated in future research. Concerning patient preference, it was observed that this point was not systematically taken into account when setting up deprescribing plans. It should therefore also be assessed in greater depth during future implementation.

Most HCPs felt that the intervention could be integrated into routine practice, but the question of appropriate remuneration was raised once, to ensure long-term implementation (maintenance). Financial incentives for deprescribing interventions have already been highlighted as a factor facilitating successful implementation.^39^^,^^51^^,^^64^

Some service adaptations have been implemented without impacting the core elements of the service. However, we observed that some full or partial medication reviews have been performed outside the intervention group, thus potentially contaminated the control group. This highlights the added value of the implementation evaluation in interpreting the trial results and underscores the challenges of maintaining strict separation between intervention and control conditions in pragmatic, real-world settings such as nursing homes. Nevertheless, we are unable to quantify this contamination, therefore the impact on the effectiveness outcome. Adaptation is often considered essential to the implementation of an intervention, as long as the core of the intervention is consistent.^68^ Furthermore, adaptations, as identified in the literature, can even improve implementation, i.e. to involve residents more in the deprescribing process,^69^ to clearly define the role of each HCP at the start of the process^14^^,^^70^ and to provide ongoing rather than one-off training for HCPs.^71^

Despite their general satisfaction with the intervention, only a few HCPs judged its impact to be positive. Change in clinical practice is known to be complex and slow.^72^, ^73^, ^74^ This can be perceived as a barrier by HCPs, even if changes take place gradually. HCPs' beliefs and attitudes regarding the implementation of new practices should be closely monitored by researchers as part of future implementation. Specific strategies should then be put in place to HCPs view change as a slow but continuous process.

The intervention was considered sustainable, as most HCPs expressed their intention to repeat it and felt that its integration into routine practice was possible. However, we observed that it was not systematically maintained one year after the intervention, suggesting that the intention to act is not sufficient to ensure the maintenance of the intervention, as the action does not occur spontaneously. Furthermore, the relatively short implementation period (October 2018–June 2019) likely limited the integration of the intervention into routine nursing home practices. Sustainability may also be affected by factors such as workload, time constraints, staff turnover (particularly among nurses), and the absence of dedicated remuneration for pharmacists' involvement. In terms of implementation strategies, pharmacist training was received positively, suggesting that it was useful for implementing the intervention. None of the participating pharmacists sought clinical support. This may be explained by the fact that, as voluntary participants, they could be considered “early adopters”,^75^ being probably more experienced than other pharmacists. As such, they would not have needed or benefited from support. This may be reinforced by the fact that healthcare professionals already had prior experience of deprescribing activities. Ongoing support has been suggested as an important implementation strategy^40^ and so, combined with the potentially biased sample, we suggest that this strategy be pursued in future implementation efforts and further evaluated.

Strengths and limitations.

The use of the implementation science framework and outcomes is a strength of this study, as it enabled multiple implementation outcomes to be explored at different stages of implementation (preparation, operation and sustainability). Indeed, the FISpH framework provide a useful structure to evaluate the implementation of a pharmacist-led service, but usually in community pharmacies. Indeed, nursing homes represent a setting in which additional contextual elements could play a role and the more recent conceptual framework for designing deprescribing studies in the nursing home setting^76^ could be more suitable for further study. One limitation of the study is that the sample size for the qualitative interviews was too small to reach thematic data saturation but the sample size was limited by the sample size of the RCT study. However, we were able to interview at least two different HCPs in each participating nursing home, which enabled us to gain an overview of the experiences of intervention providers. There may be a memory bias for interviewees, as some interviews took place several months after the intervention. Implementation was mostly evaluated at only one point in time, i.e. at the end of the intervention, via interviews with HCPs. To better evaluate implementation, we suggest adding audits and feedback throughout the tested intervention.^40^ As implementation was evaluated in the context of a randomized controlled trial, HCPs encountered some additional barriers due to regulatory requirements, such as time-consuming data collection and complex consent forms. These barriers, which are not present in current practice, made it difficult to recruit nursing home residents and may have influenced the feedback provided during interviews, as well as the implementation of the intervention. Another difficulty encountered in this implementation study was the recurrent confusion between the intervention and the study during the interviews. Finally, HCPs did not deprescribe all the PIMs detected during the study, which may pose an ethical issue. However, as the IPS service continued beyond the duration of the study, the PIMs detected may have been discussed in future rounds. In addition, while some drugs present significant potential harm, they may still be the best option for the treatment of an individual resident.

## 5 Conclusions

Pharmacist-led medication reviews on deprescribing in nursing homes were successfully implemented in the context of our pragmatic RCT, and HCPs considered its integration into routine clinical practice feasible. Certain adaptations and improvements are required to support future implementation, extension and maintenance. We recommend staggering medication reviews over the year, clearly defining and allocating the role and tasks of each HCP, integrating systematic shared decision-making with residents and their families, and strengthening interprofessional collaboration. Multiple implementation strategies, such as clinical support, specific training for pharmacists to implement the intervention, financial incentives, auditing and feedback, should be put in place and evaluated in the future.

## Funding details

This work was supported by the 10.13039/501100001711Swiss National Science Foundation, as part of National Research Program 74 “Smarter Health Care” [grant number 167509], and by the State of Vaud, through a global grant awarded to the late Prof. Olivier Bugnon and Dr. Anne Niquille.

## Authors contribution

The authors confirm contribution to the paper as follows: study conception and design: SM, JD, JM, DC, MS, and AN; project administration: SM and AN; data curation and investigation: SM and JD; formal analysis and validation: SM, JD, and JM; methodology: SM, JD, JM, DC, MS, and AN; visualization: SM, JD, JM, DC, and MS; supervision: JD and AN; funding acquisition and resources: AN. Draft manuscript preparation: SM; writing – review and editing: all authors (SM, JD, FM, JM, DC, MS, and AN). All authors approved the final version of the manuscript.

## Aknowledgments

The authors would like to thank all HCPs who took part of the implementation study. They also want to thank Nuno M. for his high-quality work on transcribing the interviews.

**Table:** Appendix 1. Interview guide synthetized for nurses, pharmacists and physicians.

Themes	Questions	Relays	Notes
Satisfaction	1.What did you think of the different stages of the project?	1’ What did you like? 1” What didn't you like?	
Pertinence	2. How do you rate the usefulness of this intervention at resident level, compared with the QC-DeMo intervention study?	2′. To what extent does the intervention proposed as part of the IDeI study meet a need on the ground?	
Improvement	3. In your opinion, which type of resident benefits most from this type of medication review? Why or why not?		
Feasibility	4. What were the practical difficulties you encountered during the intervention?	4′. Considering your current workload, to what extent do you think you could integrate this approach into routine care?	
Fidelity	5. What adaptations, if any, have you made to the approach proposed in the IDeI study?	5′. What are the reasons for this?	
Improvement	6a. What would be the ideal frequency for this type of medication review? 6b. And the ideal time?		
Improvement	7. What improvements would you suggest to the approach proposed by the IDeI study?	7′. Would you consider it possible for the medication review to be finalized in an interprofessional session? What advantages/disadvantages would you see in this?	
Improvement (pharmacists only)	8. What additional needs do you have in terms of tools, experience and training?		
Maintenance	9. Do you intend to carry out the intervention again once the study is over?		
Satisfaction	10. Generally speaking, are you satisfied with the approach proposed in the IDEI study?	□ 0 □ 1 □ 2 □ 3 □ 4 □ 5 □ 6 □ 7 □ 8 □ 9 □ 10

**Table:** Appendix 2. Summary table of determinants relating to the position and role of each healthcare profession and residents/family.

Level	Determinant	Illustrative quote
• Related to all HCPs	Classes of drugs that cannot be deprescribed	Phys _NH6: 'It's one of the most problematic classes of medication – sleeping pills, for example - people are extremely reluctant because they're afraid of losing sleep. But that's because these are very recurrent problems over a certain age ′.
	Motivation to deprescribe due to a structured study process	Phys_NH4: 'The advantage of being in a protocol like this is that (…) you're pushed to keep making efforts, because it's true that (…) afterwards you tend to give up a little (…) always fighting to get the drugs off ′.
	Interprofessional collaboration	Nurse_ NH1: 'It's the very basis of drug deprescribing… it's an effective triumvirate between physician, nurse and pharmacist. Otherwise, we can say we forget. ′ Nurse_NH6: 'What I found easy here was the involvement of the pharmacy, the pharmacist, in our institution, even before [the intervention] (…)′. Pharm_NH3: 'It depends (…) on the situation, but (…) it's basically the nurse and the physician. And then I intervene from time to time (…) if I notice that there's something (…) inconsistent in the medication. But at first, it's really the physician, the nurse and the family (…). And then afterwards, I sometimes intervene if (…) if there's a need to change something, (…) to adapt.′ Phys_NH6: 'In the end, it's easier to pass on information, the… way of working is the same, which is also a comfort for the nurses, who know what our prescribing habits are, how, in general, we intervene, and what they can ask of us'. Nurse_NH2: 'It's always (…) the physician who decides in the end. So even if we suggest something, if we analyze the situation, if the physician says no, that's it, it's over (…) it's frustrating (…)′. Pharm_N·H7: 'I think there needs to be more regular dialogue with the nurses about preparing the medication review; it's something we've really missed, this ability, this possibility of collaborating, (…) of preparing the review together. It's not something we've done in my nursing home, and I think it's something that would make the [medication] analysis easier, quicker, more relevant too, because all the information is available.
	Proximity to the workplace of the three healthcare professionals	Phys_NH5: 'In our hospital, which facilitates our work (…) I have the pharmacist who is in the building next door, I have the head nurse who is in the unit where I have my office on the second floor, (…) everything is made easier to do things, I don't think it's reproducible throughout the canton. ′
	Use of alternative therapies	Nurse_NH3: ′Over the last five years or so, we've developed the idea of trying alternative therapies before taking medications or to replace them.′
• Nurses related factors	Nurses turnover	Nurse_NH6: 'so here, at the moment (…), we've had a lot of changes in nurses; people are leaving (…) so we take on temporary nurses that we'd like them to stay, but they don't (…); so, involving (…), nurses in a study like this, it's necessarily people who stay for a while; and there, it was a bit difficult. ′
	Fear of the consequences of deprescribing	Pharm_NH6: 'the other [nurses] were a bit scared, they were scared because something had been taken away from Mr. X and Y, they were very scared'.
	Nurses' knowledge of residents	Pharm_NH2: 'I think they [the nurses] have a very good idea of the patient's condition, how it's progressing and everything that follows.
• Pharmacists related factors	Access to the resident's medical record	Pharm_NH7: 'We don't have access to the nursing home file at the pharmacy, I myself had to go to the nursing home with a nurse to access the information we needed'.
	Pharmacists' Knowledge of residents	Pharm_NH7: 'the problems we have as pharmacists is that we don't know the residents; we know their treatments, but we've generally never met them, and so we don't know what problems they have apart from those that are immediately accessible in the medical records that we were able to consult for the study, to carry out the medication review. ′
	Experience in conducting medication reviews	Pharm_NH1: 'It's not easy to do this work. You have to have experience to do it, because you have to do it, and then you have to get the lab data and the follow-up data, and the treatment, you have to put it all together, think it through. ′
• Physicians related factors	Resistance to deprescribing	Phys_NH6: 'I have colleagues who prescribe a lot and who wouldn't appreciate being asked to stop prescribing drugs. Because they would take it as a challenge to their practice.′
	External or referral physicians	Phys_NH6: 'And then I think that in other nursing homes that don't have this type of referring physician, where patients are followed by a number of. colleagues who only come in occasionally. It's much more complex. Phy_NH6: 'The advantage of being a referring physician in a home is that, overall, we're there regularly, all the time'.
	Psychogeriatric training	Phys_NH1: ′Let's just say that we're much more fortunate in a canton like Vaud or Geneva to have many more trained geriatricians, and therefore geriatricians who consult in nursing homes. And that certainly improves the quality of care.′
	Coordination between physicians in the nursing home	Phys_NH3: 'In our nursing home, there are two physicians, so a general practitioner who looks after all the somatic medication, and I look after all the psychotropic treatments. That's ‘why there was this specificity in our nursing home of saying that everyone was responsible for their own sector somewhere.′
• Residents/ Families related factors	Family involvement	Nurse_NH7: 'But there's always the difficulty of the families, (…) it's really a problem (…) because sometimes they don't have the time, they're not always available, they agree to take part but, afterwards (…) they had very little contact, I think, with the physician, the pharmacist and me (…) I didn't really get the impression that they were really involved.′
	Resident and family trust in the medical and nursing team	Nurse_NH4: 'That's when you see that they really trust us. So we have to be very vigilant, and then monitor (…) because (…) they let themselves go, and then (…) they know that we're looking after, if you like, their health and everything, and (…) often, they take their medication, but without really asking. ′
	Residents' fear/refusal to be deprescribed medications	Phys_NH4: 'when we're dealing with people who (…) are fearful or very symptomatic, there are (…) anxious people who complain of a whole host of ailments, and it's not easy to deprescribe them'. Pharm_NH6: 'There are people who have some cognitive problems or who are more or less anxious (…) it was better not to ask their opinion. But it was a problem, it was an obstacle for us.′ Inf_NH4: 'This study may upset naturally anxious people'. Phy_NH6: ′Unfortunately few residents complain about having too much medication, which is a bit surprising but it's often the case and, and sometimes it's even complicated to remove them.′
	Placebo effect	Phys_NH5: 'and this can sometimes be true through the placebo effect: if you're convinced that without this treatment you're going to be unwell, well, you're going to be unwell; for sure, the power of the psyche is going to reflect on the somatic, automatically. So I think that we can't force people and I understand the resistance (…) at this level'.
	Resident turnover	Phys_NH4: 'The big problem is that there's a fairly rapid turnover of our patients in nursing homes - the average length of stay for a patient in nursing homes is two years. So we can imagine that after two years, almost all the patients have changed, and all the deprescribing work we do will start again with the arrival of new patients.′ Phys_NH6: 'but at one time we had such a high turnover rate that we didn't really have time to do anything'.
	Reassurance from the physician	Phys_NH6: 'We tend to explain (…) to these patients that, in the end, there are a lot of things that aren't essential, and that it's not dangerous to stop anyway, and that we'll reassess the situation. The idea is to reassure people that ‘if things don't go well, don't worry, we're not going to leave you with your symptoms if they come back, with pain or discomfort that's not acceptable’′.
	Capacity for discernment	Phys_NH1: 'We have the advantage that patients no longer have the discernment to tell us what they want or don't want, so it's like with very young children in pediatrics: it's up to us, with the clinical examination, to determine what they have and give them what's most appropriate.′ Pharm_NH6: 'people who are very old, and who (…) are completely clear about their treatment; and these people, in general, when there was a request for deprescribing, they accepted it completely (…). The people for whom we were able to do it, we did it, were people who had a good grasp of their treatment.′ Nurse_ NH7: 'If the resident is capable of deciding for himself (…), there's no problem; (…) But it's especially for people who don't have the capacity for discernment.′

## Declaration of competing interest

The authors declare that they have no known competing financial interests or personal relationships that could have appeared to influence the work reported in this paper.

## Data Availability

The authors confirm that the data supporting the findings of this study are available within the article and its Appendixs 1 and 2. Raw data were generated at Unisanté. Analytic codes and data coding schemes supporting the findings of this study are available from the corresponding author SM on request.
